# Retraction: An immunoinformatics and extensive molecular dynamics study to develop a polyvalent multi-epitope vaccine against cryptococcosis

**DOI:** 10.1371/journal.pone.0322316

**Published:** 2025-04-04

**Authors:** The PLOS One Editors

The *PLOS One* Editors retract this article [1] due to concerns about peer review integrity, authorship, and potential manipulation of the publication process. These concerns call into question the validity and provenance of the reported results. We regret that the issues were not identified prior to the article’s publication.

MRSS, NAR, MMEE, MSH, MAAM, RBP, ATM, IJB, SN, IJK, SR, JAP, and JA did not agree with the retraction. ZR either did not respond directly or could not be reached.
